# Clinical Immunogenicity of rHuPH20, a Hyaluronidase Enabling Subcutaneous Drug Administration

**DOI:** 10.1208/s12248-015-9782-0

**Published:** 2015-05-13

**Authors:** Sanna Rosengren, Samuel S. Dychter, Marie A. Printz, Lei Huang, Richard I. Schiff, Hans-Peter Schwarz, John K. McVey, Fred H. Drake, Dan C. Maneval, Don A. Kennard, Gregory I. Frost, Barry J. Sugarman, Douglas B. Muchmore

**Affiliations:** Halozyme Therapeutics, Inc., 11388 Sorrento Valley Road, San Diego, California 92121 USA; Baxter Bioscience, Deerfield, Illinois USA; Baxter Bioscience, Vienna, Austria; Baxter Healthcare Corporation, Deerfield, Illinois USA

**Keywords:** anti-drug antibodies, clinical trial, immunogenicity, rHuPH20, subcutaneous drug delivery

## Abstract

Recombinant human PH20 hyaluronidase (rHuPH20) is used to facilitate dispersion of subcutaneously delivered fluids and drugs. This report summarizes rHuPH20 immunogenicity findings from clinical trials where rHuPH20 was co-administered with SC human immunoglobulin, trastuzumab, rituximab, or insulin. Plasma samples were obtained from evaluable subjects participating in ten different clinical trials as well as from healthy plasma donors. A bridging immunoassay and a modified hyaluronidase activity assay were used to determine rHuPH20-reactive antibody titers and neutralizing antibodies, respectively. rHuPH20-binding antibody populations from selected subjects with positive titers were affinity-purified and subjected to further characterization such as cross-reactivity with endogenous PH20. Among individual trials, the prevalence of pre-existing rHuPH20-reactive antibodies varied between 3 and 12%, excepting the primary immunodeficiency (PID) studies. Incidence of treatment-induced rHuPH20 antibodies was 2 to 18%, with the highest titers (81,920) observed in PID. No neutralizing antibodies were observed. Within most trials, the kinetics of antibody responses were comparable between pre-existing and treatment-induced antibody responses, although responses classified as persistent were more common in subjects with pre-existing titers. There was no association between antibody positivity and either local or systemic adverse events. Pre-existing and treatment-induced antibody populations were of similar immunoglobulin isotypes and cross-reacted to endogenous PH20 to similar extents. No cross-reactivity to PH20 paralogs was detected. rHuPH20 induces only modest immunogenicity which has no association with adverse events. In addition, antibodies purified from baseline-positive individuals are qualitatively similar to those purified from individuals developing rHuPH20-reactive antibodies following exposure to the enzyme.

**Electronic supplementary material:**

The online version of this article (doi:10.1208/s12248-015-9782-0) contains supplementary material, which is available to authorized users.

## INTRODUCTION

Hyaluronan (HA) is a mega-dalton glycosaminoglycan which constitutes a component of the extracellular matrix. Due to its viscosity and high water binding capacity, the HA viscoelastic gel forms a barrier to fluid flow and molecular movement through the interstitial collagenous matrix present in the hypodermis (1). This places a limit on the volume and composition of fluids and drugs that can be injected into the subcutaneous (SC) space. To overcome this limitation, hyaluronidases purified from bovine or ovine testes have been employed for decades to facilitate dispersion and absorption of subcutaneously injected solutions (2), but their clinical use has generally been limited by impurity profiles (1) and issues of hypersensitivity reactions (see for example (3)). More recently, the human testicular hyaluronidase (PH20, encoded by the gene SPAM1) was cloned and described as a GPI-anchored protein (4) that possesses hyaluronidase activity (5). This discovery allowed the engineering of a recombinant form of the human PH20 protein (rHuPH20) lacking the GPI anchor domain (6) which can be purified to homogeneity with a specific activity 50- to 100-fold greater than commercially available animal-derived hyaluronidase products.

The suitability of using rHuPH20 to facilitate dispersion of injected fluids and drugs was demonstrated preclinically in models employing rodents (1) as well as pigs (7–9). Clinical trials with rHuPH20 have been undertaken in the areas of rapid large volume hydration (10,11), rapid insulin and insulin analog delivery in subjects with diabetes (12–15), and delivery of large proteins such as immunoglobulins and monoclonal antibodies by SC rather than intravenous (IV) injections (9,16–21). Importantly, the use of SC rHuPH20 in combination with various drugs has been demonstrated to generate pharmacokinetic (PK) profiles that provide advantageous or comparable (i.e., noninferior) characteristics when compared to those obtained by IV injection of drug alone (16–20,22), and to increase the absorption rate of SC delivered insulin and its analogs (12,14,15) as well as morphine (23) among others. These data demonstrate the potential of rHuPH20 to facilitate the delivery of a variety of drugs in both acute and chronic clinical settings, either enabling transition to a more convenient, patient-friendly route of administration (e.g., conversion of treatments from IV to SC) or improving the PK profile of drugs that are typically administered SC (e.g., short acting insulin products).

Even though the use of human proteins as therapeutics can be expected to reduce immunogenicity relative to non-human proteins, the potential still exists of an antibody response. In fact, clinical immunogenicity of recombinant human proteins is frequently reported (24). The clinical relevance of those immune responses can vary widely and must be determined on a case-by-case basis through investigation of the impact of the immune response on product efficacy and safety. For example, anti-drug antibodies (ADA) may have significant impact on efficacy, through the development of neutralizing antibodies, and/or drug PK (25). Cross-reactivity of neutralizing antibodies with an endogenous counterpart can also occur, and in some cases this has been associated with grave clinical consequences (26). The potential impact of ADA has led to the development of regulatory guidelines for immunogenicity testing and reporting (27,28). Somewhat surprisingly, a number of cases have been reported in which pre-existing antibodies to biotherapeutics are present in a certain percentage of the population, prior to any exposure to the drug in question (29,30). The reason for these pre-existing antibodies is typically poorly understood, but their presence can complicate the interpretation of anti-drug antibody testing results obtained after administration of the drug in question.

Since rHuPH20 has been used clinically to facilitate dispersion and absorption of several co-administered biotherapeutic agents, it represents a unique opportunity to evaluate immunogenicity in multiple patient populations and dosing regimens. This report summarizes rHuPH20 immunogenicity findings from clinical trials where rHuPH20 was co-administered SC with human immunoglobulin, trastuzumab, rituximab, and insulin. In addition, a study was undertaken to determine the baseline prevalence of rHuPH20-reactive antibodies in the general population. Finally, antibodies from subjects who became positive for rHuPH20-reactive antibodies following exposure to rHuPH20 were characterized along with pre-existing antibodies from subjects who had never been treated with hyaluronidase. Results suggest that rHuPH20 induces only modest immunogenicity, with no meaningful changes to adverse event profiles. In addition, antibodies purified from individuals with pre-existing positive titers have isotypes and binding characteristics to endogenous PH20 similar to those purified from individuals developing rHuPH20-reactive antibodies following exposure to the protein.

## MATERIALS AND METHODS

### Clinical Trials

The designs of individual clinical trials for which immunogenicity analysis of rHuPH20 was conducted are described in detail in Supplemental 1. A summary of all included trials is provided in Table I.

**Table I:** Overview Over All Included Clinical Trials

Trial	Clintrials.gov reference	Population	Co-administered therapeutic	rHuPH20 dose	rHuPH20 dosing frequency	Duration of rHuPH20 treatments	Duration of available immunogenicity data
117–203	NCT00883558	Type I diabetes mellitus	Prandial insulin	Variable	3 times daily	3 months	Up to 6 months
117–205	NCT01194245	Type I diabetes mellitus	Prandial insulin analog	75 (12–63) μg daily^*a*^	3 times daily	12 weeks	Up to 6 months
117–206	NCT01194258	Type II diabetes mellitus	Prandial insulin analog	240 (22–1460) μg daily	3 times daily	12 weeks	Up to 6 months
117–403	NCT01848990	Type I diabetes mellitus	Continuous SC insulin infusion	150 U per treatment event	Every 2–3 days	Up to 21 months	183 (30–183) days
HannaH	BO22227, NCT00950300	HER2-positive breast cancer	Trastuzumab	10,000 U per treatment event	Every 3 weeks	18 cycles of 3 weeks each	724 (85–944) days
SparkThera	BP22333, NCT00930514	Follicular lymphoma	Rituximab	23,400 U per treatment event	Every 2–3 months	Up to 2 years (stage 2)	85 (17–646) days
SABRINA	BO22334, NCT01200758	Follicular lymphoma	Rituximab	23,400 U per treatment event	Every 8 weeks	Up to 2 years	315 (116–842) days
SAWYER	BO25341, NCT01292603	Chronic lymphocytic leukemia	Rituximab	26,740 U per treatment event	Once per 5 of 6 cycles (stage 2)	Up to 2 years	133 (28–399) days
160603/902	160603: NCT00814320 160902: NCT01175213	Primary immunodeficiency	Human IgG	2800 (800–6400) U per treatment event	Every 3–4 weeks	Up to 3 years	1414 (337–1599) days
Normals	n/a	Healthy plasma donors	n/a	n/a	n/a	n/a	Single sample

^*a*^When applicable, data are presented as median (min–max)

### Sample Collection

Blood samples, anticoagulated with EDTA, were obtained at the time of clinic visits according to the individual trial schedules. When relevant, samples were obtained immediately prior to the next scheduled treatment of rHuPH20 in order to avoid drug interference effects. Upon centrifugation, the resulting plasma was stored at approximately −20°C and transported frozen to the testing laboratory.

### Assays for rHuPH20-Reactive and Neutralizing Antibodies

In order to detect antibodies to rHuPH20, an electrochemiluminescence (ECL)-based bridging immunoassay was developed and validated according to recent regulatory guidelines (27,28) and white papers (31,32). After an overnight co-incubation of plasma sample diluted 1:5 with rHuPH20 conjugated to biotin and rHuPH20 conjugated to Sulfo-TAG (250 ng/mL each; Meso Scale Discovery, Rockville, MD), the resulting immune complex was captured onto streptavidin-coated plates and detected in a SECTOR 2400 instrument using ECL Read buffer (all Meso Scale Discovery). A three-tiered approach was employed consisting of screening, specificity testing using unconjugated rHuPH20 as a competitor, and two-fold step-wise titering in negative base pool plasma diluted 1:5. Statistically based cut points for screening positivity, specificity, and titration were established as recommended in (32). Individual sample titers were defined as the last dilution that yielded a positive response. The positive control for this method was a rabbit anti-rHuPH20 antibody affinity-purified from a pool of serum from three rabbits immunized with rHuPH20 in Freund’s adjuvant. In order to determine assay sensitivity, a range of positive control concentrations was spiked into human plasma and evaluated using the bridging immunoassay to determine the threshold response *versus* the established screening cut point. While some of the ECL responses at 50 pg/mL fell above the assay cut point, all of the responses at 150 pg/mL were demonstrated to be greater than the cut point, and hence this conservative value was chosen to represent assay sensitivity. Taken into consideration the 1:5 dilution of plasma, the sensitivity of this assay was thus determined to be ≤750 pg/mL, a threshold that was 660-fold greater than the recommended 500 ng/mL for screening assays (28).

In the case of the HyQvia study, primary immunodeficiency (PID) subjects were treated with pools of human IgG, which were shown to contain low levels of rHuPH20-reactive antibodies. This is a reflection of the baseline prevalence of anti-rHuPH20 in the general population described in “Results” section. Consequently, subjects in this study who were identified as not being able to produce mature antibodies due to their underlying immunodeficiency syndrome (X-linked agammaglobulinemia, severe combined immunodeficiency, or hyper IgM syndrome) nonetheless presented with rHuPH20-reactive antibody titers ranging from 10 to 80, which were interpreted as the result of passive transfer of the antibodies contained in the therapeutic agent. Accordingly, HyQvia subjects were only considered to have a positive rHuPH20-reactive antibody response if a sample titer was ≥160.

An assay for neutralizing antibodies (nAb) against rHuPH20 was based on the USP assay for hyaluronidase activity (33). Briefly, plasma samples diluted 1:20 were pre-incubated with 2 U/mL rHuPH20 for at least an hour and was then allowed to digest high molecular weight hyaluronan for 30 min. Addition of acidified serum resulted in turbidity at 640 nm due to the presence of precipitated hyaluronan which was monitored spectrophotometrically; any neutralizing antibody in the plasma sample diminished the capability of rHuPH20 activity to lower the turbidity. The minimum required plasma dilution of 1:20 was determined based on the potential for interference by known plasma components such as inter-α-inhibitor (34).

For reporting immunogenicity responses, terms such as antibody prevalence, incidence, pre-existing and treatment-induced antibodies, kinetics (transient *vs.* persistent), and titer increase over baseline (treatment-boosting) were defined as in (35). In addition, the requirement for an increase of two or more titering steps in order to consider a baseline-positive subject treatment-boosted was also defined as in (35). In this case, since the titering took place in 2-fold steps, that meant that a 4-fold or higher increase in titer was required for such classification.

### Adverse Events Analysis

For trial 160603/902, each reported adverse event in subjects that developed rHuPH20-reactive antibodies was graded mild, moderate, or severe, and the number of adverse events occurring prior to and following the first positive titer were added for these subjects and expressed per time unit to yield an adverse events rate.

### rHuPH20-Reactive Antibody Purification

For further characterization, rHuPH20-reactive antibodies were affinity-purified from 130 to 250 mL plasma from four HyQvia subjects who had previously yielded ECL bridging immunoassay titers in excess of 10,000 and who had titers of 2560 to 10,240 at the time of providing the sample used as a source for antibody. As controls, 700–800 mL obtained by plasmapheresis of four healthy volunteers with rHuPH20-reactive antibody titers of 160–640 was included. For affinity chromatography, Sepharose 4 Fast Flow resin (GE Healthcare, Pittsburgh, PA) was conjugated with rHuPH20 and packaged into Vantage chromatography columns (EMD Millipore, Billerica, MA) connected to an ÄKTA Purifier instrument (GE Life Sciences, Piscataway, NJ). Each plasma sample was diluted with 4 vol of TBS and loaded onto a fresh column, and antibodies were eluted with 0.1 M glycine-HCl buffer, pH 2.5, and immediately neutralized. Following dialysis against PBS, samples were concentrated to 0.5–1 mL using 30 kDa molecular weight cut-off concentrators.

### Antibody Isotyping

The resulting purified antibody preparations were isotyped using a Human/Non-Human Primate isotyping kit (Meso Scale Discovery), and IgG subclasses were determined using a Human IgG subclass profile ELISA for IgG1-IgG3 (Life Technologies), and separately using a Human IgG4 ELISA (eBioscience). Within each preparation, the amount of each isotype/subclass was expressed as a percentage of the total amount of antibody.

### Evaluation of Antibody Cross-Reactivity

Endogenous PH20 was released from human sperm (Fairfax Cryobank, Fairfax, VA) by PI-PLC (Sigma-Aldrich, St. Louis, MO) treatment after capacitation with calcium ionophore A23187 (Sigma-Aldrich). Recombinant human PH20 was also released by PI-PLC treatment from DG44-CHO cells stably transfected with a full-length cDNA construct, and affinity-purified using Sepharose 4B conjugated to a rabbit polyclonal anti-rHuPH20 antibody and otherwise as above. The concentration of human PH20 in both preparations was determined using a sandwich ECL assay using the rabbit polyclonal antibody as a capture antibody in ECL High Bind plates (Meso Scale Discovery) and a Sulfo-TAG conjugate of the same antibody as a detecting antibody. Plates were read in a SECTOR 2400 instrument and concentrations determined using four-parameter logistic regression on ECL signal obtained using a dilution series of rHuPH20 as a standard.

In order to compare the binding of rHuPH20-reactive antibodies to rHuPH20 and endogenous full-length human PH20, plasma samples confirmed positive for rHuPH20-reactive antibodies were diluted to yield an ECL signal around 5000 units (between 10- and 40-fold final dilution) and subjected to a version of the ECL bridging immunoassay described above wherein rHuPH20 conjugates were used at 100 ng/mL. In these experiments, increasing concentrations of unconjugated rHuPH20, sperm-derived PH20, and CHO-derived full-length PH20 were included to compete for binding to rHuPH20-reactive antibodies present in the sample. The resulting ECL signal/log competitor concentration curves were analyzed by four-parameter logistic regression where the top and bottom asymptotes were constrained as the ECL value obtained from the un-competed sample mean and the negative control mean, respectively, and the resulting IC50 value was reported.

To determine whether treatment-induced antibodies from subjects who participated in the HyQvia trial were capable of cross-reacting to recombinant human hyaluronidase (Hyal)1 and Hyal2, plasma samples from trial 160603/902 diluted 1:50 were assayed using the ECL bridging immunoassay described above in the presence of unlabeled test antigens added as competitors at a single concentration of 10 μg/mL. Insulin glulisine and rHuPH20 were used as a negative and positive control competitors, respectively.

## RESULTS

### Baseline Prevalence and Incidence of rHuPH20-Reactive Antibodies

The baseline prevalence of rHuPH20-reactive antibodies prior to exposure to the recombinant protein is summarized for each clinical trial in Table II and varied between 3.3 and 12.1%, except for the 160603/902 (primary immunodeficiency) trial where the baseline prevalence was only 1/87. This single antibody-positive subject had previously been exposed to rHuPH20 in an earlier trial where immunogenicity was not monitored, and hence the provenance of this titer (pre-existing or treatment-induced) is not known. In a study of 961 healthy plasma donors between the ages of 18 and 65, the prevalence of rHuPH20-reactive antibodies was 5.8%, similar to those observed in the various disease populations. There was no significant difference in prevalence among men and women (34/450 *vs*. 22/455, Fisher’s exact *p* = 0.13), and no association between rHuPH20-reactive antibody positivity and age.

**Table II:** Prevalence and Incidence of rHuPH20-Binding Antibodies in All Clinical Trials

Trial	Prevalence at baseline	Treatment-induced (# subjects)	Treatment-boosted (# subjects)	Total incidence
117–203	2/46^*a*^ (4.3%)	1	0	1/40 (2.5%)
117–205	13/117^*b*^ (11.1%)	3	2	5/113 (4.4%)
117–206	4/120^*b*^ (3.3%)	2	0	2/116 (1.7%)
117–403	38/456^*c*^ (8.3%)	21	3	24/335 (7.2%)
HannaH	22/290 (7.6%)	26	10	36/290 (12.4%)
SparkThera	11/185 (5.9%)	2	4	6/185 (3.2%)
SAWYER	13/107^*d*^ (12.1%)	3	3	6/96 (6.3%)
SABRINA	28/257^*e*^ (10.9%)	11	6	17/185 (9.2%)
*All rHuPH20 trials except 160603/902*	*131/1578 (8.3%)*	*69*	*28*	*97/1360 (7.1%)*
160603/902	1^*f*^/87^*b*^ (1.1%)	14	1	15/83 (18.1%)
Normals	56/961 (5.8%)	n/a	n/a	n/a

Definitions of prevalence, incidence, treatment-induced, and treatment-boosted as in (35). The italic text signifies the summation of all preceding lines

^*a*^Including 6 subjects where only a baseline sample was available

^*b*^Including 4 subjects where only a baseline sample was available

^*c*^Including 114 subjects randomized to receive standard continuous subcutaneous insulin infusion treatment, without rHuPH20, for the period studied; as well as 7 subjects randomized to receive a treatment regimen containing rHuPH20 but where only baseline and early termination samples were available

^*d*^Including 11 subjects where only a baseline sample was available

^*e*^Including 65 subjects randomized to the IV arm, as well as 7 subjects where only a baseline sample was available

^*f*^Note that this subject had previously participated in a trial of rHuPH20 and human IgG; however, during this previous trial immunogenicity monitoring was not performed. Hence, whether this subject was positive prior to any treatment with rHuPH20 is not known

The incidence of rHuPH20-reactive antibodies following rHuPH20 exposure is also shown in Table II and is defined as the percentage of subjects that were negative at baseline but who became positive following exposure (“treatment-induced”) plus the percentage of subjects that were positive at baseline and who had a 4-fold or higher increase of titer following exposure (“treatment-boosted”), as outlined in (35). In all cases, the number of treatment-induced subjects was greater than or similar to the number of treatment-boosted subjects. The overall incidence varied from 1.7 to 18.1%.

Samples testing positive for rHuPH20-reactive antibodies were assayed for their ability to neutralize rHuPH20 enzyme activity. No instance of neutralizing antibodies was found in any sample from any of these clinical trials.

### Magnitude of rHuPH20-Reactive Antibody Responses

**Fig. 1:**
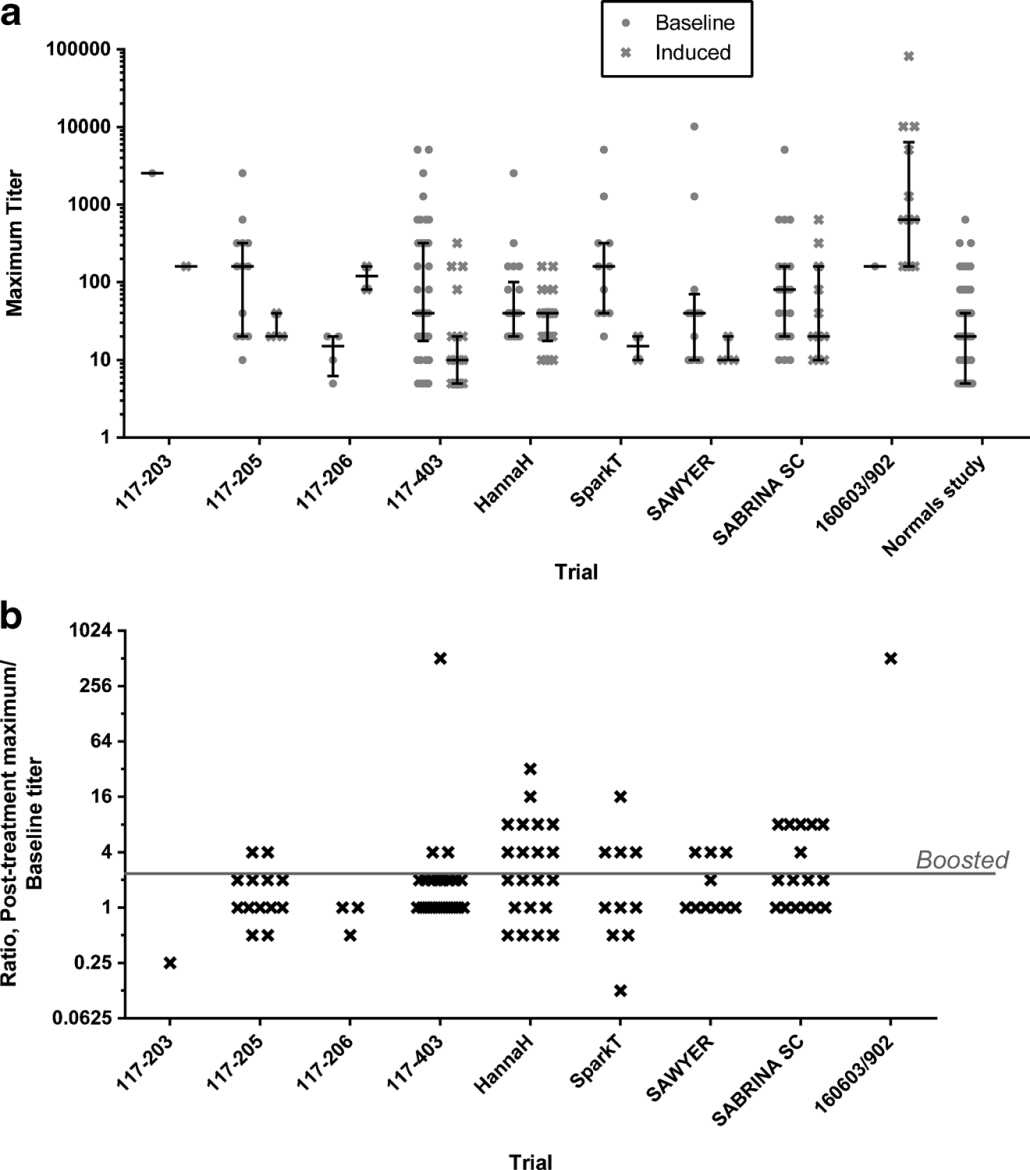
**a** Maximum rHuPH20-reactive antibody titers observed prior to rHuPH20 exposure (“Baseline”) and in subjects first testing positive following rHuPH20 exposure (“Induced”). Observations for individual subjects with median and interquartile range are indicated. **b** Maximum fold titer increase in baseline-positive subjects following rHuPH20 exposure. Individual subject observations are indicated. Subjects with more than a 2-fold titer increase following rHuPH20 exposure were considered treatment-boosted (threshold indicated by *gray line*). Ten baseline-positive subjects that were never positive following rHuPH20 exposure are not included in this figure

**Fig. 2:**
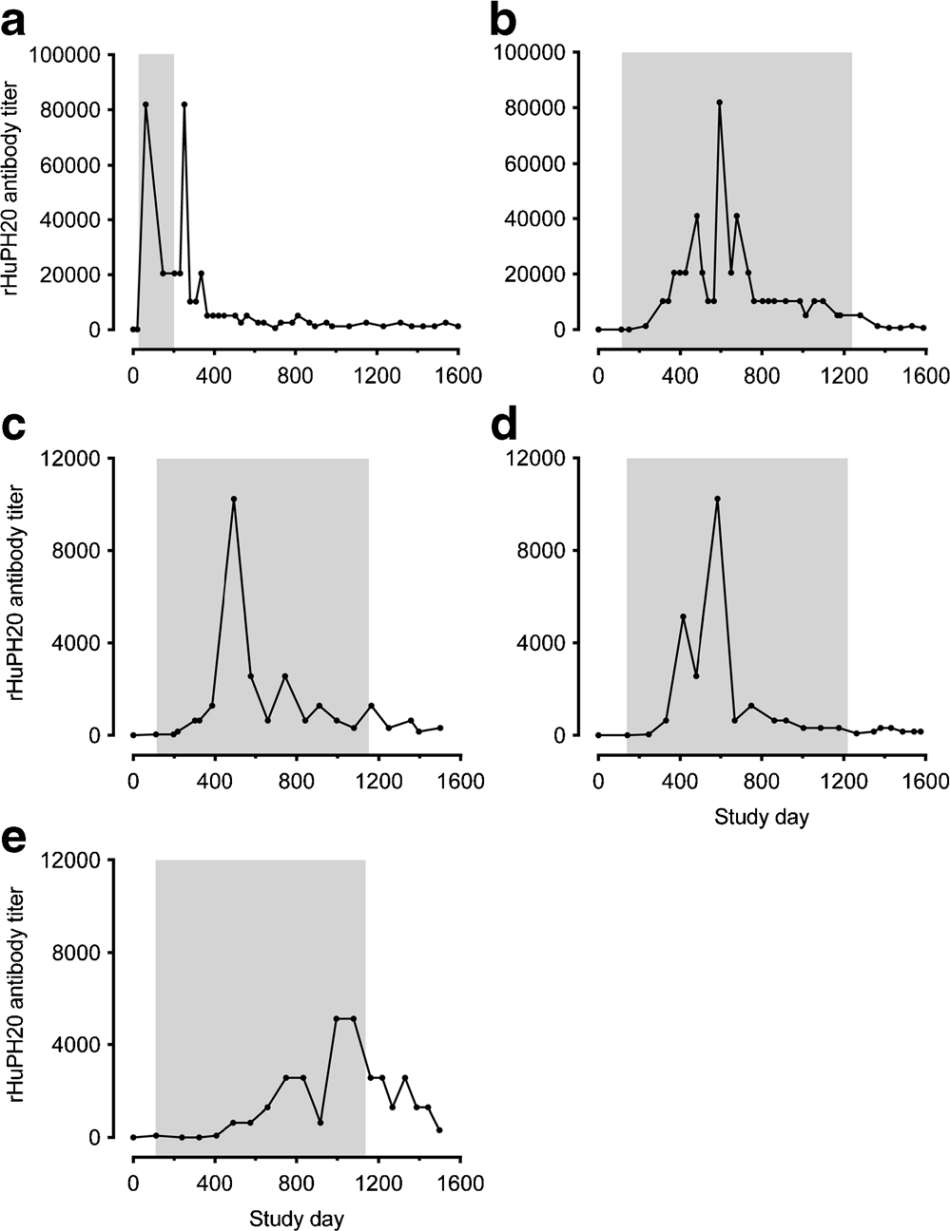
Individual rHuPH20-binding antibody titers over time in the five subjects from trial 160603/902 who had maximum titers in excess of 5000. The *shaded area* in each panel indicates the period of rHuPH20 exposure. The titers of the single subject with a positive titer (of 160) prior to participating in this study are shown in panel **a**

Titers of rHuPH20-reactive antibodies in the individual clinical trials are shown in Fig. 1a. When all studies were combined (excluding the plasma donor survey study), the median maximum rHuPH20-reactive antibody titer in subjects with positive titers at baseline and subjects who became antibody-positive *de novo* following exposure to rHuPH20, respectively, was 40 (range 5–10,240) and 40 (range 5–81,920). Generally, maximum titers of baseline and treatment-induced antibodies within each population were of similar magnitude, with two exceptions: Trial 117–206 (type II diabetes, co-administered with insulin) where the maximum titer increased following exposure, but was still only at a median of 160, and trial 160603/902 (primary immunodeficiency, co-administered with human IgG) where the highest titers in all of the trials were observed in five subjects (Fig. 2).

In the baseline-positive subjects, the fold change following exposure (Fig. 1b) did not indicate systematic titer boosting by rHuPH20, but some subjects (enumerated in Table II and shown in Fig. 1b above the gray threshold line) were considered treatment-boosted using the definition in (35). When all trials were considered together, treatment-boosting occurred in 29 of 110 evaluable baseline-positive subjects, whereas 71 baseline-positive subjects remained positive but not treatment-boosted, and ten baseline-positive subjects were not positive at any time following rHuPH20 exposure. The overall median fold titer change from baseline at post-exposure maximum was 2 (range 0.125–512). The highest extent of titer boosting was observed in one subject in trial 160603/902, who had previously been exposed to rHuPH20 (see above), as well as in one subject in trial 117–403 who had a baseline titer at 5 and a titer at 12 months of 2560.

While the highest rHuPH20-binding antibody titer responses were observed in the 160603/902 trial, titers rapidly peaked and then decreased despite continued exposure to rHuPH20. Figure 2 depicts the longitudinal time *versus* titer profiles for five subjects over the course of the study who had maximum antibody titers in excess of 5000. The subject previously exposed to rHuPH20 (panel a) had a rapid anti-rHuPH20 antibody response, and rHuPH20 exposure in this subject was stopped out of an abundance of caution for further evaluation while antibody titers were monitored over time. All other subjects (panels b–e) completed the rHuPH20 treatment period. Typically, titers peaked after study day 450 and then reverted back down while the subjects continued to receive rHuPH20 treatments.

### Kinetics of rHuPH20-Reactive Antibody Responses

**Fig. 3:**
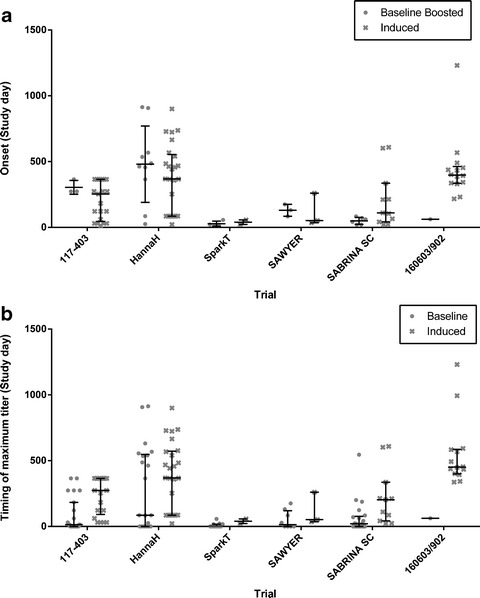
**a** Onset of rHuPH20-reactive antibody boosting (see definition in legend for Fig. 1) in subjects testing positive for antibodies prior to treatment with rHuPH20 (“Baseline Boosted”) and in subjects first testing positive following rHuPH20 exposure (“Induced”). **b** Timing of maximum rHuPH20-reactive antibody titers in subjects testing positive for antibodies prior to treatment with rHuPH20 (“Baseline”) and subjects first testing positive following rHuPH20 exposure (“Induced”). Individual observations with median and interquartile range are indicated

Evaluations of the kinetics of antibody responses were possible using data sets from six clinical trials where the period of antibody monitoring exceeded 16 weeks, or approximately 5 half-lives of IgG, in at least some of the subjects in the trial (35). The timing of the onset of antibody positivity (in subjects negative at baseline) and of the first sign of treatment boosting (in subjects positive at baseline) is summarized in Fig. 3a and is given as study day without taking the overall duration of the study into account, nor the frequency of rHuPH20 administration, so comparison between trials is not particularly informative. However, within trials, the onset of *de novo* antibody positivity and of antibody boosting was comparable, with one exception: the single baseline-positive subject in 160603/902 (who was previously exposed to rHuPH20, see above) had a very rapid onset of titer boosting compared to the timing of antibody positivity onset in the baseline-negative subjects in this trial. The timing of antibody titer maxima (Fig. 3b) also displayed a comparable pattern for baseline-positive subjects and for subjects with treatment-induced antibodies within each trial, except for trial 160603/902.

**Fig. 4:**
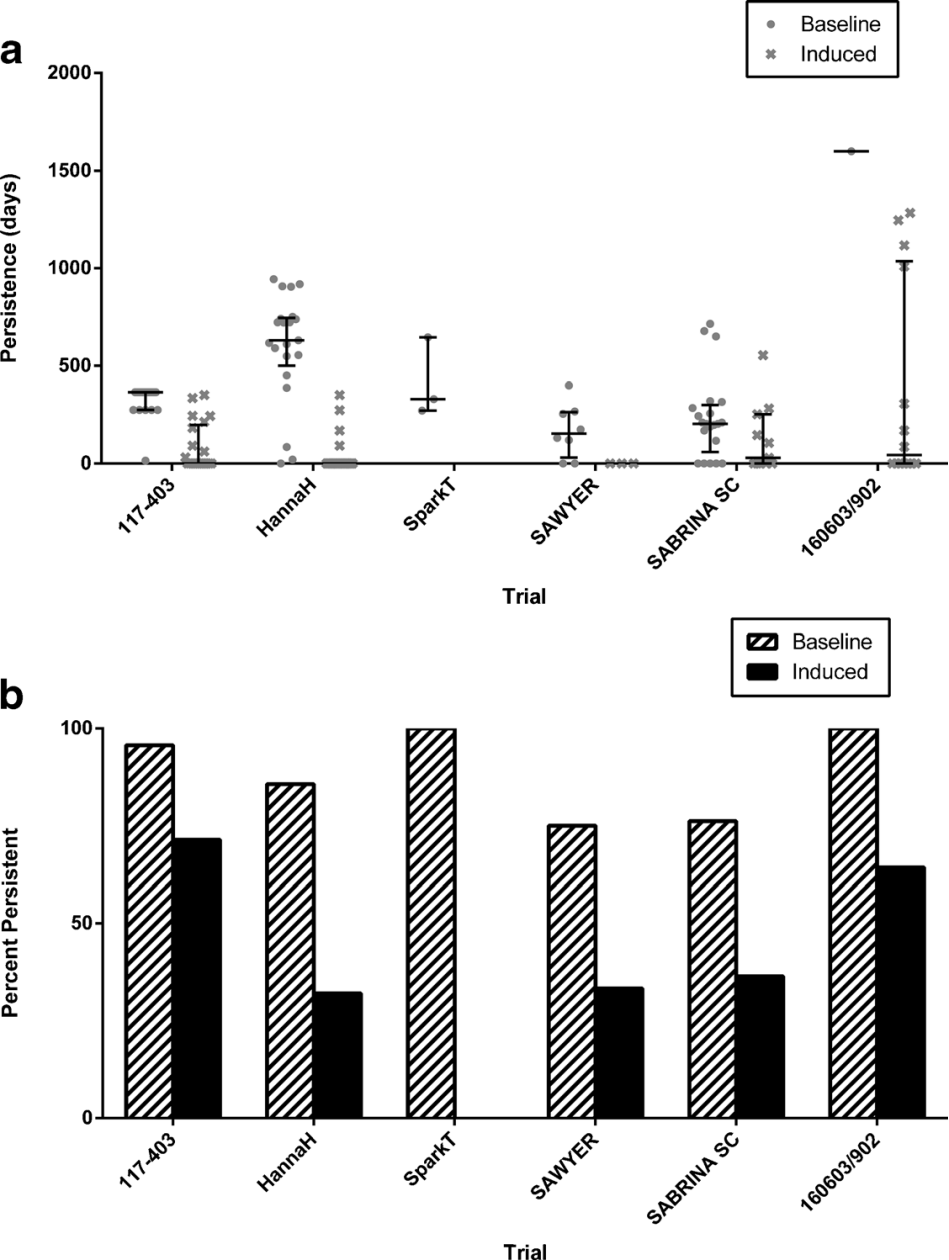
Persistence of rHuPH20-reactive antibodies in subjects testing positive for antibodies prior to treatment with rHuPH20 (“Baseline”) and subjects first testing positive following rHuPH20 exposure (“Induced”). **a** Individual observations with median and interquartile range are indicated. **b** Percentage of subjects with “Persistent” antibodies according to the definition in (35) (first and last antibody-positive samples, irrespective of any negative samples in between, are separated by a period of 16 weeks or longer, or first antibody-positive sample is obtained less than 16 weeks before last sample obtained or end of study)

Persistence of antibody positivity was determined in all subjects where the period of antibody monitoring exceeded 16 weeks. As seen in Fig. 4a, the duration of antibody positivity was generally longer in subjects who tested positive for antibodies before rHuPH20 exposure, except in the SABRINA trial where considerable overlap was observed. When the definitions for “persistent” antibody responses were applied according to (35) (see Fig. 4 legend for definition), the percent of antibody-positive subjects having persistent antibodies was always higher in the baseline-positive population (75 to 100%) than in the treatment-induced population (0 to 25%) (Fig. 4b).

### Lack of Association Between rHuPH20-Reactive Antibodies and Adverse Events

In order to determine whether any association between the conversion to antibody positivity and adverse events could be detected, data from trial 160603/902 were analyzed for all subjects who developed antibodies following rHuPH20 exposure (Table III). Results indicate that time-adjusted rates of both local and systemic adverse events classified as mild, moderate, and severe were comparable prior to and following first positive titer, with a slight decrease in each adverse event rate following rHuPH20-antibody positivity. The similarity of adverse event rates pre- and post-rHuPH20 antibody positivity also held true when the 15 subjects from trial 160603/902 were analyzed as a subset, or when the five subjects with the highest titers from this trial were considered on their own.

**Table III:** Summary of Adverse Events^*a*^ in Baxter Trials in Subjects Who Developed Anti-rHuPH20 Antibodies

Adverse events data set	Severity	Before first positive anti-rHuPH20 titer		After first positive anti-rHuPH20 titer	
		Number of events	Rate^*b*^	Number of events	Rate
Total adverse events	Mild	72	7.14	202	8.73
	Moderate	56	5.55	55	2.38
	Severe	10	0.99	9	0.39
	Total	138	13.69	266	11.50
Systemic adverse events	Mild	49	4.86	135	5.84
	Moderate	35	3.47	51	2.20
	Severe	7	0.69	9	0.39
	Total	91	9.02	195	8.43
Local adverse events	Mild	23	2.28	67	2.90
	Moderate	21	2.08	4	0.17
	Severe	3	0.30	0	0.00
	Total	47	4.66	71	3.07

^*a*^Adverse events excluding infections

^*b*^Rate = number of adverse events divided by number of years in the respective observation period, which includes 3683 and 8449 total cumulative subject days (10.08 and 23.13 total cumulative subject years) of observation prior to and following the first exposure to rHuPH20, respectively

### Characterization of rHuPH20-Reactive Antibodies

**Fig. 5:**
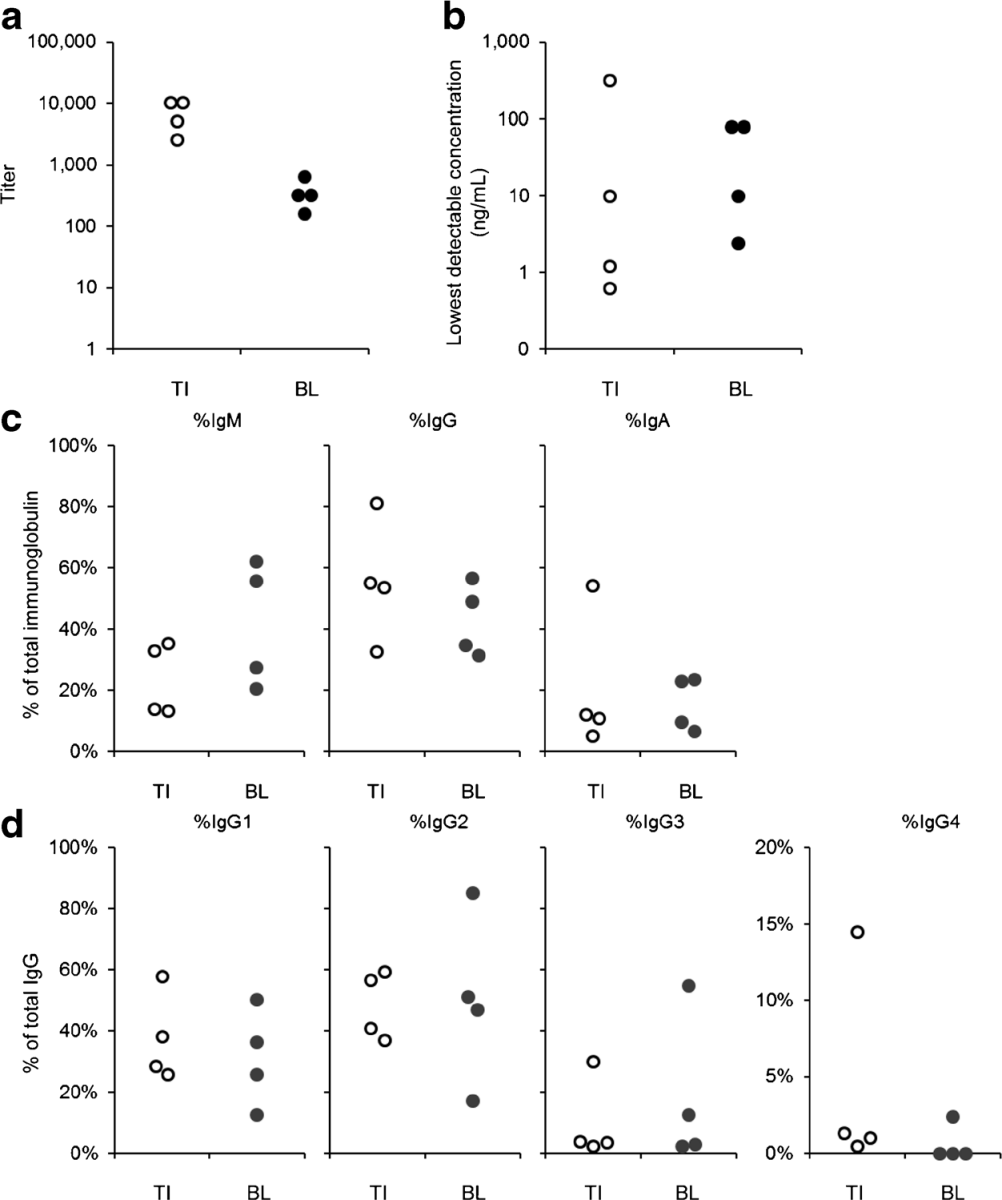
Purification and characterization of rHuPH20-reactive antibodies from PID subjects with *de novo* positive antibody titers following exposure to rHuPH20 (treatment-induced, “TI”) as well as from the baseline-positive population with pre-existing rHuPH20 antibodies (baseline, “BL”). **a** Plasma titers at the time of antibody purification. **b** Lowest detectable concentration of purified rHuPH20-reactive antibodies in the ECL bridging assay. **c** Isotypes. **d** IgG subclasses. Observations from individual antibody preparations from four individuals each are indicated

In order to compare the characteristics of rHuPH20-reactive antibodies that emerged following treatment with rHuPH20 (trial 160603/902, panels a–c and e in Fig. 2) and those present in baseline-positive individuals, rHuPH20 affinity chromatography was used to purify antibody preparations from subjects with titers at the time of plasma donation shown in Fig. 5a. Following purification, the lowest antibody concentration that could be detected using the bridging immunoassay was highly variable but within similar ranges for the two populations (Fig. 5b). Isotyping demonstrated that the two types of antibody preparations contained comparable proportions of IgM, IgG, and IgA (Fig. 5c), and percentages of individual IgG subclasses were also similar with the exception of a single elevated IgG4 level in the treatment-induced group (Fig. 5d).

**Fig. 6:**
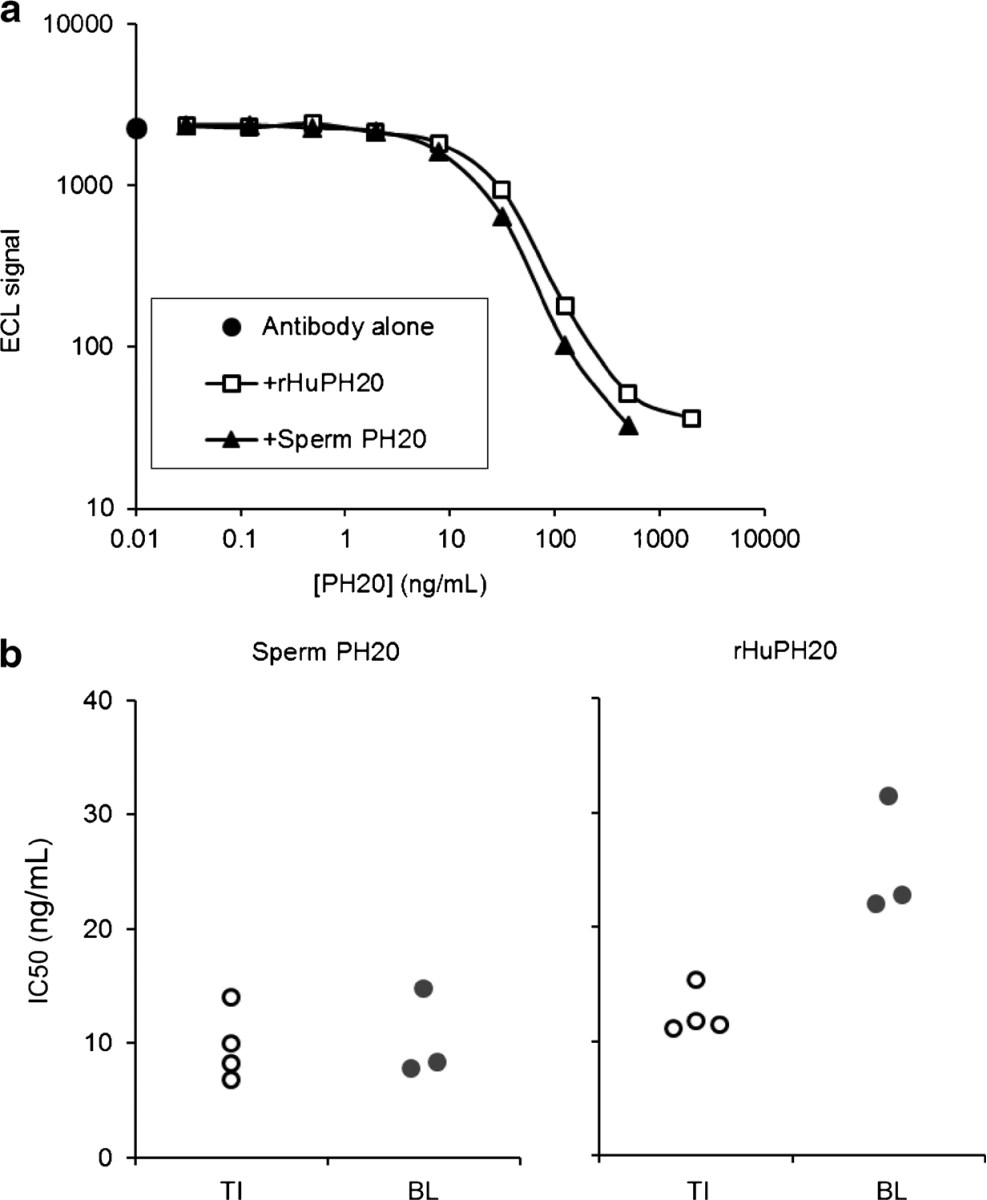
Antibody cross-reactivity to endogenous PH20. **a** Representative example of results obtained in the ECL bridging competition assay with an individual rHuPH20-reactive antibody preparation when increasing concentrations of rHuPH20 or PH20 isolated from human sperm were included. **b** Resulting IC50 values calculated from individual antibody preparations. *Open circles*, rHuPH20-reactive antibodies from PID subjects with *de novo* positive antibody titers following exposure to rHuPH20. *Closed circles*, rHuPH20-reactive antibodies from the baseline-positive population

Cross-reactivity of rHuPH20-reactive antibodies to related endogenous proteins, i.e., human sperm-derived PH20 and paralogous human hyaluronidase proteins, was studied because of the shared sequence homology between rHuPH20 and these other hyaluronidases (100% between rHuPH20 and endogenous PH20 with the exception of the C-terminal truncation, and between 34 and 42% homology with hyaluronidase (Hyal)-1, -2, -3, and -4 (36)). This evaluation was performed using a modification of the bridging immunoassay, where unlabeled proteins were used to compete in the bridging reaction. The data indicate that endogenous PH20 yielded similar results whether treatment-induced and pre-existing antibody preparations were tested (Fig. 6a), whereas rHuPH20 tended to inhibit bridging reactions with treatment-induced antibody preparations more efficiently than those with pre-existing antibodies (Fig. 6b). Under these circumstances, the highest concentration of rHuPH20 tested (2 μg/mL) inhibited the bridging signal observed with both treatment-induced and pre-existing antibodies by >99%.

In separate experiments, plasma samples from four 160603/902 subjects with the highest titer of rHuPH20-binding antibodies (as shown in Fig. 2a–d), as well as two subjects deemed antibody-negative, were evaluated for cross-reactivity to other human hyaluronidases (Hyal-1 and Hyal-2) as determined by competition by unlabeled protein at 10 μg/mL in the bridging immunoassay. In these studies, unlabeled rHuPH20 inhibited the bridging reaction between the biotinylated- and ruthenylated-rHuPH20 by greater than 99% in all samples except for the two negative samples (<23%). In contrast, the percentage inhibition for recombinant Hyal-1 and Hyal-2 as well as the glulisine negative control was less than 23% for all samples.

## DISCUSSION

The recombinant hyaluronidase, rHuPH20, is used to facilitate SC delivery of protein therapeutics that would normally require intravenous infusion and can also be used to intentionally improve the PK profile of drugs that are usually administered by SC route. It is typically co-infused SC along with the therapeutic, either using sequential administration or using co-formulated drug product. In either case, rHuPH20 allows the therapeutic agent to permeate more readily through the SC space and gain access to the central circulation via either the capillaries, for small molecule therapeutics, or the lymphatics, for large molecule therapeutics. This report summarizes the clinical immunogenicity responses to rHuPH20 in several clinical trials, including rHuPH20 administered in combination with insulin, therapeutic antibodies, or human IgG to a total of 1526 subjects from diverse populations. Overall, exposure to rHuPH20 was associated with emergence of rHuPH20-reactive antibodies in 3 to 18% of treated individuals, depending on the trial. Of the 83 individuals who were antibody-negative prior to treatment and developed rHuPH20-binding titers after exposure to the protein, only three experienced maximum titers in excess of 10,000 using a highly sensitive bridging immunoassay. In addition, 28 subjects with pre-existing rHuPH20-reactive antibodies prior to exposure experienced treatment boosting, as defined by an increase in titer of two titering steps or more following treatment, and one of these experienced a maximum titer of 81,920, the highest titer observed following rHuPH20 exposure in this data set. Importantly, no neutralizing antibodies were identified in any of these trials.

The clinical importance of ADA responses can manifest itself in various ways: neutralizing antibodies can alter drug efficacy (25) and, in the worst case, can cross-neutralize the endogenous counterpart (26); both binding and neutralizing antibodies can have an impact of the PK of the drug (25); and circulating ADA-drug immune complexes can form with potential deleterious safety consequences (37). In the case of rHuPH20, no potential impact on function of endogenous PH20 should be expected since no neutralizing antibodies were observed. No clinical data are yet available regarding the potential effect of anti-rHuPH20 antibodies on PK of rHuPH20 itself; however, the action of rHuPH20 is localized to the SC space and no evidence of systemic exposure can be detected at the doses used (17). This might be related to the rapid elimination of rHuPH20 from the central compartment. Even when infused IV in human volunteers, rHuPH20 has a terminal elimination half-life of only 5–6 min (manuscript in preparation). Finally, with regards to immune complex formation, whereas IgG can theoretically distribute to the skin and lymph (38), there was no difference in adverse event rates in subjects who eventually became positive for rHuPH20-binding antibodies when periods prior to and following first positive titer were compared.

A consistent finding through all the trials was the presence of rHuPH20-binding antibodies in a small percentage of subjects prior to rHuPH20 exposure. This was confirmed in a separate survey study of 961 healthy plasma donors where the prevalence of pre-existing antibodies was determined to be 6%. The basis for why they are sometimes present remains unknown but may be related to immune reactivity to endogenous PH20, a GPI-anchored hyaluronidase normally present on the apical head of male sperm (4,5). Whatever their provenance, the presence of pre-existing rHuPH20-reactive antibodies does not appear to be associated with systematic titer boosting after exposure to rHuPH20 in the majority of subjects. Importantly, there were no adverse events associated with anti-rHuPH20 reactivity, with or without boosting.

The comparison of several clinical trials with unique study designs, dose levels of rHuPH20, and sampling schedules can be fraught with difficulty, which can limit the conclusions that can be drawn. In the current case, the various trials yielded similar results in terms of the nature of the rHuPH20 immunogenicity response: First, within trials, pre-existing and treatment-induced antibody titers were similar, as were their kinetics of onset of positivity *versus* onset of boosting as well as time to maximum titer. Second, if treatment-induced rHuPH20 antibody responses occurred, they tended to be more transient in nature than for pre-existing antibodies. The reason for this is currently unknown, but could possibly be related to the existence of a robust and long-lasting immune response to PH20 in individuals with pre-existing antibodies which predisposed towards more durable boosting. No data exists to date to address this question. Third, no neutralizing antibodies were observed in any clinical study.

The notable exception to this pattern was the response observed in the 160603/902 trial, where rHuPH20 was administered in combination with human IgG in subjects with primary immunodeficiency and induced a durable rHuPH20-binding antibody response with titers in excess of 5000 in five subjects. These titers generally diminished despite continuing treatment, and no neutralizing antibodies were ever observed; however, the nature of these responses was different from those observed in other clinical trials with rHuPH20. The reason for this could possibly be sought in the design of the trials (e.g., dosing frequency, total dose) or the co-administered therapeutic, in this case human IgG purified and pooled from plasma donors, which could contain low levels of rHuPH20-reactive antibodies due to the prevalence of such antibodies in the general population as described above. This latter notion was confirmed by analysis of preparations of human IgG which contained approximately 0.01–0.02% of rHuPH20-reactive antibodies (unpublished observations). Besides these differences, another potential reason for the differential immunogenicity response to rHuPH20 in the 160603/902 trial was the subject population. Primary immunodeficiencies constitute a diverse group of disorders of monogenic or polygenic origin, where the genetic basis of the disorder in the more frequently diagnosed disorders such as CVID is frequently unknown (39). Many of these diseases predispose affected subjects to dysregulated T-cell tolerance and autoimmunity (40,41), including CVID (42), which was diagnosed in three of the five cases in which the highest rHuPH20-reactive antibody titers were observed.

Beside the possibility that antibodies to rHuPH20 could attenuate *in vivo* enzymatic activity and produce a loss of dispersive effect at the injection site, the other theoretical concern is whether these antibodies could affect the functionality of endogenous PH20 or closely related enzymes. *In vitro* binding of anti-rHuPH20 antibodies to endogenous PH20 would be expected because of the high homology between rHuPH20 and PH20. On the other hand, binding of such antibodies to PH20 *in vivo* would be limited by the limited tissue expression profile of PH20 (adult male reproductive tract) as well as the blood-testis and blood-epididymal barriers limiting access of antibodies in systemic circulation to that milieu (43).

In order to address some of these concerns, treatment-induced rHuPH20-reactive antibodies as well as those from baseline-positive subjects were affinity-purified using rHuPH20-coupled resin and extensively characterized using various methods such as antibody titer, antibody isotype, and *in vitro* cross-reactivity to endogenous human PH20 and related hyaluronidases. The resulting antibody preparations from rHuPH20-treated and baseline-positive subjects had similar isotypes and IgG subclasses, suggesting that the degree to which immune class switching had taken place was comparable in both populations. Cross-reactivity analysis demonstrated that both types of antibody preparations bound to endogenous PH20 *in vitro* to a similar degree, whereas the treatment-induced antibodies bound somewhat better to rHuPH20 than did the pre-existing ones. This indicates that rHuPH20-binding antibodies emerging following treatment, even in the case of the comparably high titers occasionally observed in the 160603/902 trial, share molecular and immunological characteristics with those observed in more than 5% in the general population.

With respect to other human hyaluronidases, the risk of anti-rHuPH20 antibodies binding to them is lower because the primary structure of rHuPH20 is only distally related (i.e., 34 to 42% homology) to Hyal-1, -2, -3, and -4 (36). In support of this notion, none of these plasma samples cross-reacted to recombinant Hyal-1 or Hyal-2 when using the modified bridging immunoassay. This conclusion is corroborated by results from a tissue cross-reactivity study using the affinity-purified anti-rHuPH20 antibodies to stain different human tissues (unpublished observations); the only specific immunohistochemical staining observed was in the seminiferous tubules in the testis. This expression profile differs from that observed for other hyaluronidases, which are expressed in many tissues including lung, liver, skeletal muscle, and kidney (44). If anti-rHuPH20 antibodies cross-reacted with other hyaluronidase family members, multiple tissues would have been expected to be positive in the tissue cross-reactivity study. Thus, the lack of signal in multiple assay systems indicates that anti-rHuPH20 antibodies did not cross-react with PH20 paralogs.

These observations serve to alleviate potential concerns raised by the apparent binding (although not neutralization) of a treatment-induced antibody to an endogenous protein involved in aspects of reproduction and are further supported by published reports in which several attempts were made to immunize males with PH20 as an immunocontraceptive approach in animal models. These studies involved rabbits (45,46), mice (47), and guinea pigs (48), and only the latter experienced infertility following PH20 immunization with a crude testicular extract that resulted in autoimmune orchitis (49). Furthermore, sperm from mice lacking PH20 were able to fertilize eggs, albeit in a somewhat delayed manner (50).

In conclusion, rHuPH20 is a recombinant human protein that appears to have modest immunogenicity and no deleterious effects on efficacy or adverse events. In addition, antibodies that bind to rHuPH20 are present in about one in 20 healthy individuals not exposed to the protein, and the functional characteristics of pre-existing and treatment-induced antibodies are similar. Therefore, rHuPH20 continues to constitute an attractive therapeutic option for delivering large molecules and fluid volumes via the SC route as an alternative to IV administration.

## Electronic supplementary material

Supplemental 1 (DOCX 26 kb)
