# Supplementary material for: Clinical Immunogenicity of rHuPH20, a Hyaluronidase Enabling Subcutaneous Drug Administration
Source: AAPS J. 2015 May 13;17(5):1144–56. doi: 10.1208/s12248-015-9782-0 (PMC4540732; doi:10.1208/s12248-015-9782-0)
Supplement: Supplementary file 1 — (DOCX 26 kb) [file 12248_2015_9782_MOESM1_ESM.docx]

# SUPPLEMENTAL 1

## Detailed description of included clinical trials

### Insulin trials with rHuPH20 in Type 1 and Type 2 diabetes

Immunogenicity data from three clinical trials in Type 1 diabetes mellitus (Studies 117-203/ NCT00883558, 117-205/ NCT01194245, and 117-403/ NCT01848990) and one trial in Type 2 diabetes (Study 117-206/ NCT01194258) are included in this report. Study 117-203, a two-way cross-over study originally described in (1), was designed to test for noninferiority of prandial insulin-PH20 compared to prandial insulin lispro (both injected SC) with respect to glucose profiles. Following a one-month titration period during which subjects were trained to optimize basal and prandial insulin dose titration, subjects were randomized to receive three-month treatment cycles of insulin-PH20 followed by insulin lispro, or vice versa. Patients titrated their doses of test articles to achieve tight postprandial glycemic control. The insulin-PH20 solution contained rHuPH20 at 5 µg/mL, but insulin and rHuPH20 doses during treatment were not recorded.

Study 117-205 and study 117-206, both two-way cross-over studies, were essentially designed the same: prandial Lispro-PH20 or Aspart-PH20 (collectively described as Analog-PH20) was compared to prandial insulin lispro alone with respect to glycemic control as determined by change in glycosylated hemoglobin as well as postprandial glucose profiles. Subjects received approximately 3 daily SC injections of insulin lispro or Analog-PH20, each administered over the course of two sequential 12-week treatment periods with the treatment sequence randomized. Based on treatment records, the median daily dose (range) of rHuPH20 was approximately 77 (12-268) U in study 117-205, and 245 (22-1489) U in study 117-205.

For these three diabetes studies, plasma samples for immunogenicity monitoring were obtained at baseline and at the end of each treatment period. Subjects in 117-203 had an additional plasma sample obtained at the end of the titration period, prior to randomization into treatment groups.

Study 117-403 was a 4-arm hybrid crossover/parallel group controlled study in subjects who use rapid acting analog insulin (RAI) delivered by means of continuous SC insulin infusion (CSII). Subjects were randomized 1:1:1:1 to receive either (1) a commercially available preparation of rHuPH20 (*Hylenex*) or (2) a precommercial formulation of *Hylenex*, delivered as an SC pretreatment each time an infusion set is deployed (every 2-3 days) for the full 24 month duration of the study; or to receive either (3) CSII treatment using *Hylenex* SC pretreatment each time an infusion set is deployed for 12 months followed by crossover to standard CSII treatment for the next 12 months or (4) standard CSII treatment for 12 months followed by crossover to *Hylenex* SC pretreatment each time an infusion set is deployed for the next 12 months. Both formulations of rHuPH20 are administered at 150 U per pretreatment event. Immunogenicity data from the first 12 months of this trial are included herein.

### Subcutaneous trastuzumab with rHuPH20 in HER2-positive breast cancer

The HannaH study has been described previously (2). It was a randomized, open-label study (NCT00950300) in which subjects with HER2-positive breast cancer were assigned to trastuzumab administered either IV, or SC along with rHuPH20. Trastuzumab+rHuPH20 was first administered over 8 cycles every 3 weeks, concomitant with chemotherapy (docetaxel for the first 4 cycles, then fluorouracil, epirubicin, and cyclophosphamide for the second 4 cycles). Patients then underwent surgery, and continued to receive trastuzumab+rHuPH20 therapy every 3 weeks for 10 additional cycles, along with hormonal therapy and radiotherapy according to local practise, resulting in a total of 18 trastuzumab+rHuPH20 cycles. Subjects received a fixed dose of rHuPH20 at each SC injection event of 10,000 U. Samples for immunogenicity assessment were collected at baseline, on day 1 of cycles 2, 5 (pre-surgery), 13 and 18 (post-surgery) and at months 3, 6, 12, 18 and 24 following the last trastuzumab dose.

### Subcutaneous rituximab with rHuPH20 in follicular lymphoma

Immunogenicity data from two trials with rituximab SC (combined with rHuPH20) in subjects with follicular non-Hodgkin’s lymphoma, both of which are outlined elsewhere (3), are reported here. The first (SparkThera, NCT00930514) was a Phase Ib study in two stages; in Stage 1, rituximab SC was administered once, followed by maintenance therapy on rituximab IV every 2-3 months for up to two years, in order to determine the dose level for pharmacokinetic noninferiority to rituximab IV at 375 mg/m^2^. In Stage 2, subjects were randomized to rituximab SC (fixed dose of 1400 mg in 11.7 mL containing rHuPH20 at 2000 U/mL) or rituximab IV (375 mg/m^2^) administered on day 1 during maintenance cycles every 2-3 months. Plasma samples for immunogenicity testing were obtained at baseline, around day 20 of the first cycle, immediately prior to the second cycle (day 57 or 85), and 3 and 9 months following the last dose.

Data from an ongoing Phase III study (SABRINA, NCT01200758) are also included. This is a a two-stage, randomised, controlled, open-label, phase III study of patients with previously untreated follicular lymphoma administered induction chemotherapy plus either rituximab SC 1400 mg as above, or rituximab IV 375 mg/m^2^. All subjects receive rituximab IV at cycle 1 and are then randomized into SC or IV groups. Subjects responding to treatment after 8 cycles are eligible for maintenance treatment with rituximab SC or rituximab IV monotherapy every 8 weeks for 2 years. Plasma samples are obtained prior to dosing in each treatment cycle and every 12 weeks until 96 weeks after the last rituximab dose.

### Subcutaneous rituximab with rHuPH20 in chronic lymphocytic leukemia (CLL)

An ongoing Phase 1b trial (SAWYER, NCT01292603) of rituximab SC in previously untreated CLL is outlined in (3). This two-stage trial was designed to determine the pharmacokinetics and safety of rituximab SC in combination with fludarabine and cyclophosphamide. In Stage 1, subjects received a single dose of rituximab SC (1400 mg, 1600 mg, or 1870 mg, all in dosing solutions containing rHuPH20 at 2000 U/mL) following 5 treatment cycles (28 days each) with rituximab IV and pharmacokinetic parameters were determined. Based on these data, a dose of 1600 mg of rituximab SC was selected for ongoing Stage 2 testing wherein one cycle of rituximab IV is followed by 5 cycles of rituximab SC.

### Subcutaneous human immunoglobulin with rHuPH20 in primary immunodeficiency (PID)

This prospective, open-label study (160603, NCT00814320) investigated the efficacy and tolerability of rHuPH20-facilitated human immunoglobulin SC (IGHy) in patients with PID. The study design as well as efficacy, safety, and pharmacokinetics results have been described previously (4). Subjects initially received IGHy for approximately 14-18 months, at each individual subject’s prestudy frequency of intravenous human immunoglobulin (IGIV). rHuPH20, at a dose of 75 U/g IgG, was administered through a SC needle, followed by IgG 10% at 108% of the pre-study IGIV dose calculated on a weekly equivalent basis, via the same SC needle. This dosing regimen resulted in a median (range) rHuPH20 exposure of 2800 (800-6400) U every 3-4 weeks (median frequency 4 weeks). Plasma samples for immunogenicity monitoring were obtained at baseline and then at an individualized frequency up to immediately prior to each other infusion event (i.e. every 6-8 weeks), with the sampling frequency decision based on each subject’s positivity for rHuPH20-reactive antibodies and the occurrence of adverse events suspected to be associated with rHuPH20 treatment.

All subjects were eligible to enroll in a long-term extension safety and tolerability study (160902, NCT01175213) using the same treatment schedule as in the original trial for up to 5 years, however this trial was halted in July, 2012 over concerns of rHuPH20 immunogenicity. In this extension study, plasma samples for immunogenicity monitoring were obtained every 12 weeks.

### Normal volunteer study

In order to determine the baseline prevalence of rHuPH20-reactive antibodies in the general population, a study was undertaken of healthy individuals volunteering to donate blood for a plasma bank. Target enrollment was about 1,000 individuals equally divided between men and women, age 18-65. Informed consent specific to this study was obtained in addition to the plasma donation consent.

# REFERENCES FOR SUPPLEMENTAL 1

1. Vaughn DE, Muchmore DB. Use of recombinant human hyaluronidase to accelerate rapid insulin analogue absorption: experience with subcutaneous injection and continuous infusion. Endocr Pract. 2011;17(6):914-21.

2. Ismael G, Hegg R, Muehlbauer S, Heinzmann D, Lum B, Kim SB, et al. Subcutaneous versus intravenous administration of (neo)adjuvant trastuzumab in patients with HER2-positive, clinical stage I-III breast cancer (HannaH study): a phase 3, open-label, multicentre, randomised trial. Lancet Oncol. 2012;13(9):869-78.

3. Shpilberg O, Jackisch C. Subcutaneous administration of rituximab (MabThera) and trastuzumab (Herceptin) using hyaluronidase. Br J Cancer. 2013;109(6):1556-61.

4. Wasserman RL, Melamed I, Stein MR, Gupta S, Puck J, Engl W, et al. Recombinant human hyaluronidase-facilitated subcutaneous infusion of human immunoglobulins for primary immunodeficiency. J Allergy Clin Immunol. 2012;130(4):951-7 e11.
